# Case report and comprehensive literature review: A rare instance of lipoid pneumonia

**DOI:** 10.1097/MD.0000000000047537

**Published:** 2026-02-06

**Authors:** Yang Liu, Man Wang, Shi-Huan Yu

**Affiliations:** a Department of Respiratory Medicine, The First Affiliated Hospital of Harbin Medical University, Harbin, Heilongjiang Province, China.

**Keywords:** endogenous lipoid pneumonia, exogenous lipoid pneumonia, lipoid pneumonia

## Abstract

**Rationale:**

This case report elucidates the diagnostic challenges and natural progression of lipoid pneumonia (LP), a rare pulmonary condition, highlighting the critical importance of long-term monitoring.

**Patient concerns:**

An 82-year-old female presented with a 2-year history of chronic cough and an acute exacerbation.

**Diagnoses:**

Initial diagnosis was severe pneumonia. Definitive diagnosis of LP was confirmed by computed tomography -guided lung biopsy, which revealed lipid-laden macrophages.

**Interventions:**

Suspected lipid exposure was discontinued, and supportive conservative care was provided.

**Outcomes:**

Despite intervention, serial follow-up computed tomography scans showed slow radiographic progression of the pneumonia.

**Lessons:**

LP has nonspecific presentations, leading to underdiagnosis. Diagnosis requires suspicion, exposure history, and pathological confirmation. Management focuses on eliminating the cause and supportive care, yet progression may still occur, necessitating long-term monitoring.

## 1. Introduction

Lipoid pneumonia (LP) is an uncommon condition characterized by abnormal lipid accumulation in the lungs, with autopsy series reporting an adult incidence of 1.0–2.5%.^[1]^ LP is classified into 2 subtypes based on the source of lipids. Exogenous LP typically occurs due to the inhalation or aspiration of animal fats, mineral oils, or vegetable oils,^[2]^ whereas endogenous LP results from lipid accumulation within alveolar macrophages secondary to bronchial obstruction, chronic pulmonary infections, pulmonary alveolar proteinosis (PAP), or lipid storage diseases.^[3]^ This exogenous form is commonly used in clinical practice.

Exogenous LP can be categorized into acute and chronic forms. The acute variant results from sudden accidental aspiration of large quantities of lipid material, leading to acute lung injury. In contrast, the chronic form develops secondary to intermittent, recurrent aspiration of oily substances.^[1,4]^ This condition demonstrates a predilection for elderly patients (typically aged 60–70 years), although pediatric cases have been well-documented, particularly among children with underlying risk factors for aspiration.^[5]^

Endogenous LP originates from pulmonary-derived lipids, predominantly cholesterol and its esters. This condition primarily develops when bronchial obstruction, chronic pulmonary infections, PAP, or lipid storage diseases cause the release of lipids from alveolar walls. These lipids are subsequently phagocytized and accumulated by alveolar macrophages.^[3]^

In this report, we present the case of an 82-year-old female patient with a 2-year history of cough that worsened over 2 weeks before admission. Arterial blood gas analysis revealed type I respiratory failure, and chest computed tomography (CT) revealed bilateral pulmonary infiltrates. When empirical treatment proved ineffective, bronchoscopy was performed, but showed no significant abnormalities. A definitive diagnosis of LP was established through CT-guided percutaneous lung biopsy. Following discharge, the patient discontinued lipid exposure and underwent regular follow-up examinations; however, gradual disease progression was still observed in accordance with the case report reporting guidelines.

## 2. Case report

**Figure 1.**
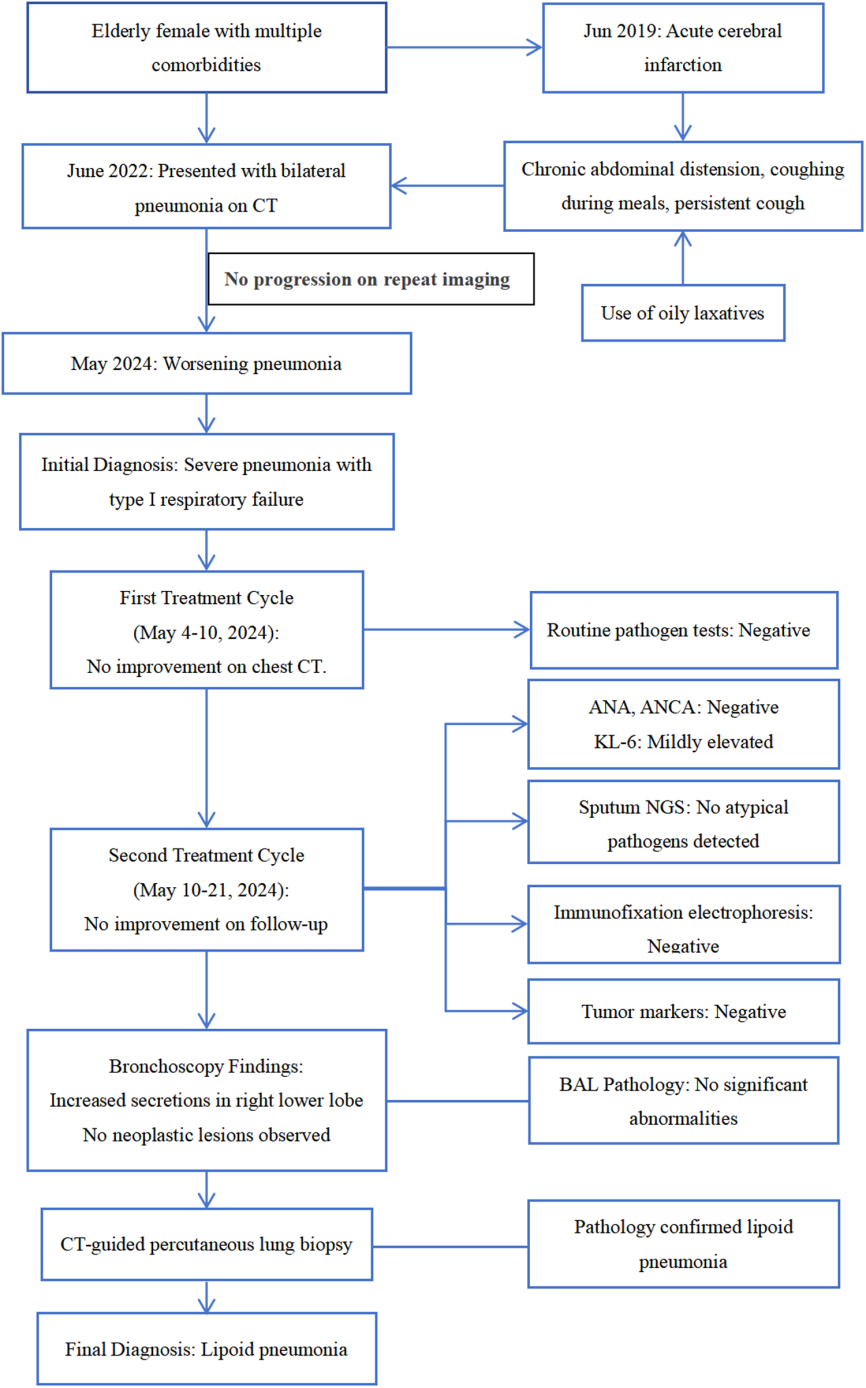
Diagram of the patient’s diagnosis and treatment process (see the glossary for abbreviations used in this article). ANA = antinuclear antibody, CT = computed tomography, KL-6 = Krebs von den Lungen-6, NGS = next-generation sequencing.

**Figure 2.**
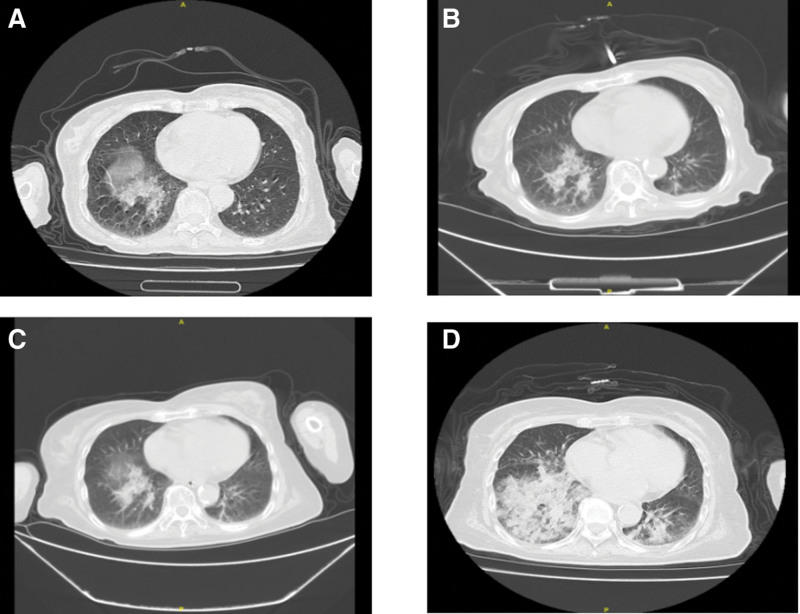
Chest computed tomography scan results of patients from June 2022 to May 2024. The results showed that multiple patchy shadows could be seen in the lower lobes of both lungs with blurred edges and uneven ends. (A) June 12, 2022. (B) December 27, 2022. (C) September 09, 2023. (D) May 04, 2024.

We report the case of an 82-year-old female with multiple comorbidities, including diabetes mellitus (complicated by bilateral diabetic foot), cerebellar atrophy, and Parkinson’s disease (Fig. 1). The patient had a history of acute cerebral infarction in June 2019, which resulted in persistent right-sided limb dysfunction and occasional episodes of dysphagia with coughing during meals. She also reported chronic abdominal distension with intermittent use of oily laxatives. On May 4, 2024, the patient sought medical attention for a 2-year history of cough that had worsened over half a month, with occasional sputum production. The patient had previously visited the hospital in June 2022 for a cough, and chest CT revealed right lower lobe pneumonia. Anti-inflammatory treatment showed no significant improvement, and subsequent regular follow-up was performed. The current admission was due to a significant progression of pneumonia (Fig. 2).

**Figure 3.**
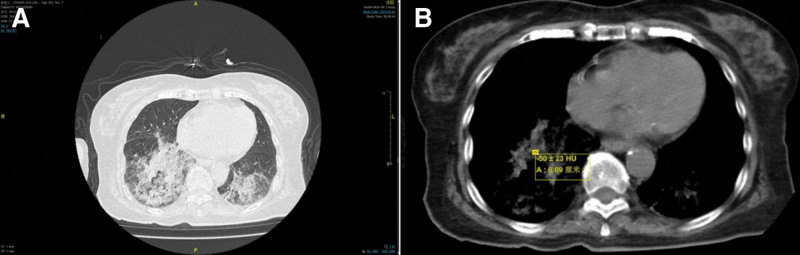
Chest computed tomography shows bilateral pneumonia lesions, manifested as ground glass opacities in the middle lobe of the right lung and lower lobes of both lungs, consolidation shadows, and thickening of interlobular septa (crazy paving) (May 04, 2024).

On admission, physical examination revealed cyanotic lips and coarse breath sounds with audible moist rales in both lungs, particularly in the right posterior chest region. Arterial blood gas analysis performed while the patient was receiving 2 L/min oxygen via a nasal cannula showed a potential of hydrogen of 7.41, partial pressure of oxygen in the artery of 61.3 mmHg, and partial pressure of carbon dioxide of 42.5 mmHg. Chest CT imaging demonstrated inflammatory changes in both lungs, characterized by ground glass opacities in the right middle lobe and bilateral lower lobes, along with areas of consolidation and interlobular septal thickening presenting the typical “crazy-paving” pattern (Fig. 3).

Laboratory tests showed: white blood cell 7.03 × 10^9^/L, C-reactive protein 10.04 mg/L, interleukin-6 12.4 pg/L, procalcitonin 0.04 ng/L, and globulin 49.18 g/L, with normal cardiac enzymes, liver/kidney function, and lipid profiles. Cardiac ultrasound revealed an ejection fraction of 56% and a normal heart structure and size. The patient scored 2 points on the confusion, urea, respiratory rate, blood pressure, age ≥65 years, and 122 points on the pneumonia severity index (class IV intermediate risk), leading to an initial diagnosis of severe pneumonia with type I respiratory failure.

**Figure 4.**
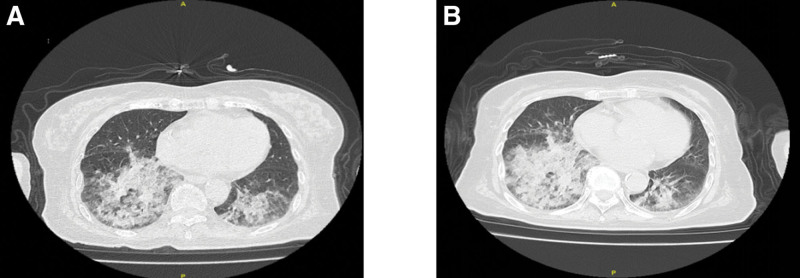
Chest computed tomography changes in the lungs of patients undergoing 1 cycle of treatment. (A) May 04, 2024. (B) May 10, 2024.

During the first treatment cycle, from May 4 to10, 2024, the patient was treated with anti-inflammatory therapy while undergoing comprehensive microbiological testing. The diagnostic workup included sputum studies for bacterial and fungal cultures as well as mycobacterium tuberculosis DNA detection; fungal biomarker tests for (1,3)-β-D-glucan and galactomannan; respiratory viral polymerase chain reaction panel covering influenza A/B, adenovirus, respiratory syncytial virus, and parainfluenza virus; and serological testing for immunoglobulin M antibodies against mycoplasma, chlamydia, legionella, adenovirus and respiratory syncytial virus. All the test results were negative. Although the patient’s systemic symptoms improved, a persistent cough with sputum production was observed, and follow-up CT revealed unresolved pulmonary inflammation (Fig. 4).

**Figure 5.**
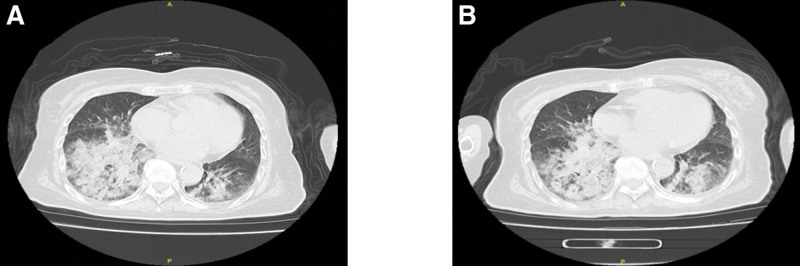
Chest computed tomography changes in the patient’s lungs at the end of the second treatment cycle. (A) May 10, 2024. (B) May 21, 2024.

During the second treatment cycle (May 10–21, 2024), considering potential noninfectious factors, the treatment regimen was adjusted, and further investigations were conducted. The patient had persistently elevated globulin levels before admission. Urine immunofixation electrophoresis returned negative results, ruling out multiple myeloma and light chain disease. Autoimmune workup, including antinuclear antibody and anti-neutrophil cytoplasmic antibody tests, was negative, while Krebs von den Lungen-6 showed mild elevation, excluding connective tissue disease-associated interstitial lung disease. Metagenomic next-generation sequencing of sputum failed to identify any atypical pathogens, thereby excluding infections caused by uncommon microorganisms. Following the 2 treatment cycles, the patient’s cough showed no improvement, and follow-up chest CT revealed persistent pulmonary inflammation without resolution (Fig. 5).

**Figure 6.**
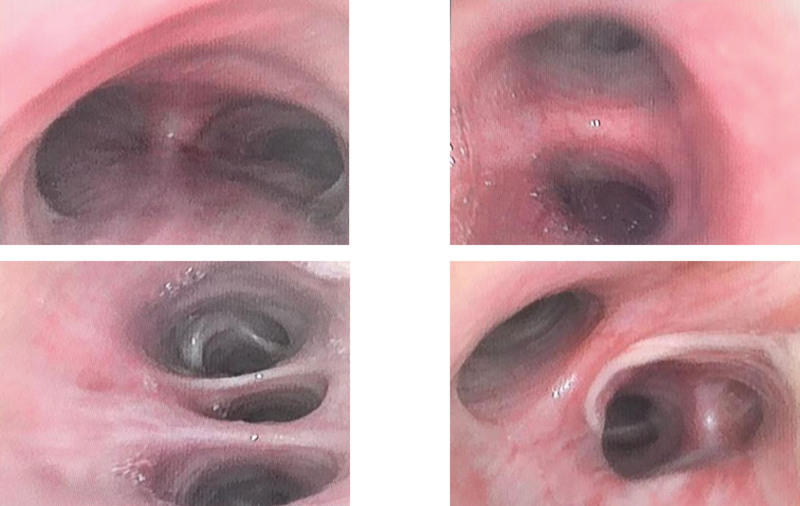
Results of fiberoptic bronchoscopy: under bronchoscopy, the bronchial openings of each lobe and segment of both lungs were normal, no abnormal tumor was found, and more secretions were observed in the lower lobe of the right lung.

The patient underwent bronchoscopy, and no new abnormal organisms were observed under a microscope. Increased secretion was observed in the lower lobe of the right lung (Fig. 6). Alveolar lavage fluid was sent for examination, but no tumor cells were found on pathological examination. Moderate numbers of neutrophils and epithelial cells and a small number of lymphocytes and phagocytes were observed, and the presence of lung malignancy was ruled out.

**Figure 7.**
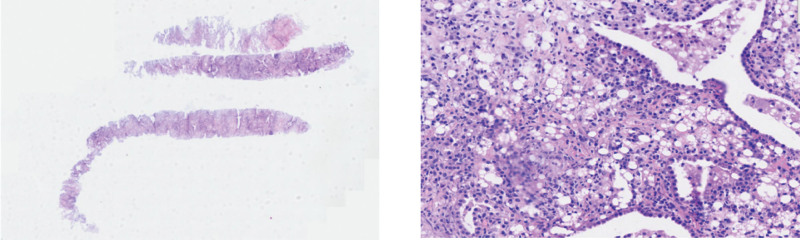
Biopsy: (right lung) part of the alveolar structure disappeared, and the alveolar septum widened. A large number of lymphocytes, plasma cells, and neutrophils infiltrated the stroma, a large number of tissue cells containing lipid vacuoles, fibrous tissue, and smooth muscle tissue hyperplasia, a small amount of carbon deposition, and alveolar epithelial hyperplasia.

A CT-guided percutaneous lung biopsy was performed, and histopathology showed partial disappearance of the alveolar structure in the right lung, widening of alveolar septa, infiltration of numerous lymphocytes, plasma cells, and neutrophils in the interstitium, abundant tissue cells containing lipid vacuoles in the interstitium and respiratory cavity, proliferation of fibrous and smooth muscle tissue, small amount of carbon deposition, and proliferation of the alveolar epithelium (Fig. 7).

**Figure 8.**
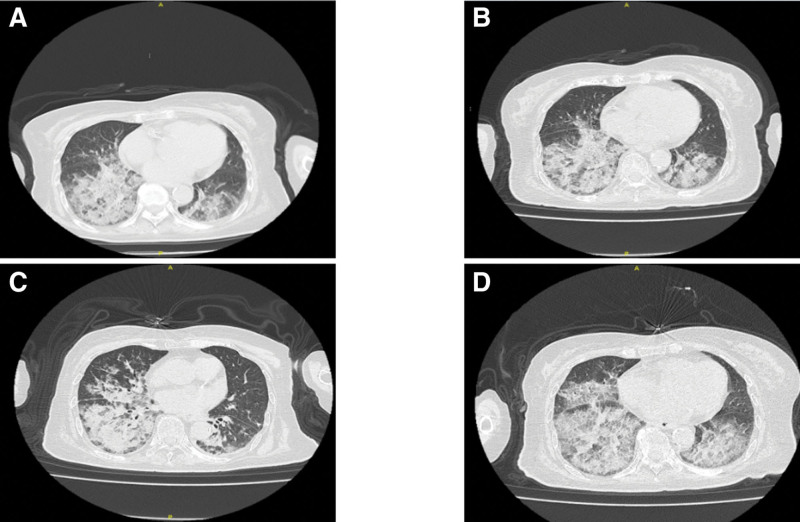
Chest computed tomography results of patients from May 2024 to March 2025: slow progression of pneumonia in patients. (A) May 27, 2024. (B) August 31, 2024. (C) November 10, 2024. (D) March 09, 2025.

Based on a comprehensive evaluation of the patient’s exposure history and diagnostic findings, a definitive diagnosis of LP was established. The clinical team recommended whole-lung lavage as the primary therapeutic intervention. However, after careful consideration of the patient’s advanced age and potential procedural risks, the family declined further aggressive treatment. Following discharge, the patient was maintained on regular outpatient follow-up with serial monitoring, which demonstrated a gradual progression of pulmonary inflammation on subsequent imaging (Fig. 8).

## 3. Discussion

LP is a rare condition caused by abnormal lipid accumulation in the lungs. They are classified into 2 types based on their lipid source: exogenous and endogenous. The exogenous type is more commonly observed in clinical practice.

Exogenous LP can be classified as either acute or chronic. The acute form typically manifests as low-grade fever, cough, chest pain, and dyspnea, whereas the chronic form is often asymptomatic and predominantly occurs in elderly patients. Clinically, patients usually present with nonspecific symptoms that frequently show discordance with imaging findings, where symptoms are mild, but radiographic abnormalities appear more pronounced. The development of chronic exogenous LP may result from mineral oil inhalation or administration (either rectally or subcutaneously, leading to oil embolism), as well as predisposing factors, including age-related pharyngeal/esophageal abnormalities, psychiatric disorders, and neuromuscular diseases causing swallowing dysfunction.^[6]^ In this particular case, the patient had multiple underlying conditions that served as risk factors, including preexisting swallowing dysfunction with occasional choking during meals, chronic abdominal distension, and long-term use of oily laxatives. Based on a comprehensive evaluation, including exposure history, imaging studies, and confirmatory lung biopsy results, the patient was ultimately diagnosed with chronic exogenous LP.

High-resolution CT is the optimal imaging modality for diagnosing exogenous LP, with the most common radiographic findings including airspace consolidation, ground glass opacities, crazy paving patterns, interlobular septal thickening, and nodular or mass-like lesions. Studies have demonstrated that low CT attenuation values ranging from −30 to −150 Hounsfield units within areas of pulmonary consolidation strongly suggest the presence of intrapulmonary fat deposition, which supports the diagnosis of LP, particularly in patients with a documented history of lipid exposure.^[6,7]^ Chest CT on admission revealed characteristic features of ground glass opacities, consolidation, and a crazy paving pattern, with notably fat-dense areas within consolidated regions. Chronic exogenous LP typically follows an indolent course, and serial imaging studies during follow-up at our institution have comprehensively documented the disease progression. Initial CT in June 2022 demonstrated ground glass opacities and consolidation predominantly in both lower lobes, which remained relatively stable during subsequent monitoring. However, the May 2024 follow-up scan showed significant progression of bilateral lower lobe lesions with new right middle lobe infiltration. Further history-taking revealed that several months before this clinical deterioration, the patient was placed in a gastric tube due to choking and coughing while eating, and accidentally inhaled paraffin oil during the placement process. This lipid aspiration event was identified as the most probable cause of the acute pulmonary deterioration observed in this patient with chronic LP.

The characteristic finding of lipid-laden macrophages in the bronchoalveolar lavage fluid (BALF) is a key diagnostic feature of LP. These macrophages are filled with lipid droplets, and the nucleus is often pushed to 1 side, giving a foamy or “wheel-like” appearance. This can be highlighted by Oil Red O or Sudan staining, which shows the lipid components as red or orange, respectively, providing diagnostic confirmation. Multinucleated giant cells may also be present in the chronic or granulomatous forms of LP, indicating chronic inflammation or granuloma formation. In addition to macrophages, BALF may contain lymphocytes, neutrophils, or eosinophils, reflecting varying degrees of the inflammatory response.^[8,9]^Patients with exogenous LP often require differential diagnosis from other conditions. In exogenous LP, BALF typically shows macrophages that phagocytize exogenous lipid droplets (exhibiting birefringence under polarized light), whereas endogenous LP is primarily characterized by cholesterol crystals (appearing as needle-shaped clefts). On tissue biopsy with special staining, exogenous LP demonstrates positive Oil Red O staining, whereas endogenous LP presents with aggregates of foam cells.^[6,10]^ In this particular case, although the BALF appeared normal, a tissue biopsy revealed numerous lipid-vacuolated histiocytes. However, the possibility of concurrent endogenous LP cannot be definitively ruled out without additional testing. Furthermore, since periodic acid-schiff staining was not performed on the BALF, potential PAP could not be ruled out.^[11]^

The treatment of exogenous LP primarily focuses on cessation of lipid exposure and supportive care. Glucocorticoid therapy may be considered for acute or subacute cases, whereas whole-lung lavage is reserved for severe diffuse disease or cases refractory to steroid treatment. In endogenous LP, management first addresses the underlying condition with glucocorticoids when significant inflammation is present, and surgical resection is considered for localized lesions. However, these treatments have limitations, and the efficacy of glucocorticoids remains uncertain owing to the lack of randomized controlled trial evidence with variable response rates and potential suppression of macrophage function that may delay lipid clearance. Whole-lung lavage is associated with significant procedural risks and may not be tolerated by some patients.^[6,8,12,13]^ Current management emphasizes avoidance of causative agents and symptomatic support, highlighting the importance of early diagnosis and timely intervention as well as the critical role of regular follow-up in monitoring disease progression and ensuring patient well-being.

Due to the patient’s advanced age, conservative treatment was adopted as the primary approach. After discontinuation of lipid exposure, follow-up chest CT scans revealed slow progression of pneumonia, which may be attributed to several factors: even after lipid exposure is stopped, lipids already deposited in the alveoli and interstitium may continue to trigger macrophage and lymphocyte infiltration, causing chronic inflammation, whereas lipid metabolites may act as antigens that induce persistent immune responses; lipid deposition may impair local defense mechanisms, leading to secondary bacterial or fungal infections; the limited clearance capacity of alveolar macrophages may result in long-term lipid retention or secondary lipid metabolism disorders, with residual lipids potentially being broken down by pulmonary enzymes into pro-inflammatory mediators that exacerbate damage; and the possible coexistence of other diseases. Therefore, if clinical deterioration persists, reevaluation of the diagnosis and adjustment of the treatment strategy may be necessary, including consideration of experimental therapies, such as whole lung lavage or even lung transplantation.

## Acknowledgments

The authors declare that financial support was received for the research, authorship, and publication of this article. This work was supported by the Heilongjiang Province “Post a Notice and Select Champions” Science and Technology Research Project (2022ZXJ03C01).

## Author contributions

**Conceptualization:** Yang Liu.

**Supervision:** Man Wang.

**Writing – original draft:** Yang Liu.

**Writing – review & editing:** Man Wang, Shi-Huan Yu.
